# Multicenter exploration of specialist palliative care in patients with left ventricular assist devices – a retrospective study

**DOI:** 10.1186/s12904-024-01563-8

**Published:** 2024-09-23

**Authors:** Theresa Tenge, Shaylin Shahinzad, Stefan Meier, Manuela Schallenburger, Yann-Nicolas Batzler, Jacqueline Schwartz, Anja Coym, Johannes Rosenbruch, Mitra Tewes, Steffen T. Simon, Carmen Roch, Ute Hiby, Christian Jung, Udo Boeken, Jan Gaertner, Martin Neukirchen

**Affiliations:** 1 Department of Anesthesiology, Medical Faculty and University Hospital Duesseldorf, Heinrich Heine University Duesseldorf, Duesseldorf, Germany; 2 Interdisciplinary Center for Palliative Medicine, Medical Faculty and Center for Integrated Oncology Aachen Bonn Cologne Duesseldorf (CIO ABCD), University Hospital Duesseldorf, Heinrich Heine University Duesseldorf, Moorenstrasse 5, Duesseldorf, 40225 Germany; 3 Palliative Care Unit, Department of Oncology, Hematology and Bone Marrow Transplant, University Medical Center Hamburg-Eppendorf, Hamburg, Germany; 4 Department of Palliative Medicine, LMU University Hospital, LMU Munich, Munich, Germany; 5 Department of Palliative Medicine, University Hospital Essen, University of Duisburg-Essen, Essen, Germany; 6 Department of Palliative Medicine and Center for Integrated Oncology Aachen Bonn Cologne Duesseldorf CIO ABCD, Faculty of Medicine and University Hospital, University of Cologne, Cologne, Germany; 7 Interdisciplinary Center for Palliative Medicine, University Hospital Wuerzburg, Würzburg, Germany; 8 RHÖN-Klinikum AG, Campus Bad Neustadt, Bad Neustadt an Der Saale, Germany; 9 Department of Cardiology, Pulmonology and Vascular Medicine, Medical Faculty and University Hospital Duesseldorf, Heinrich-Heine-University Duesseldorf, Duesseldorf, Germany; 10 Department of Cardiac Surgery, Medical Faculty and, University Hospital Duesseldorf, Heinrich-Heine-University Duesseldorf, Duesseldorf, Germany; 11 Palliative Care Center Basel, Basel, Switzerland; 12 Department of Clinical Research, University of Basel, Basel, Switzerland

**Keywords:** Palliative medicine, Heart failure, Heart-assist devices, Quality of life, Retrospective studies, Multicenter study

## Abstract

**Background:**

The number of advanced heart failure patients with left ventricular assist devices (LVAD) is increasing. Despite guideline-recommendations, little is known about specialist palliative care involvement in LVAD-patients, especially in Europe. This study aims to investigate timing and setting of specialist palliative care in LVAD-patients.

**Methods:**

We conducted a retrospective multicenter study in 2022. Specialist palliative care services in German LVAD-centers were identified and invited to participate. Forty adult LVAD-patients (mean age 65 years (SD 7.9), 90% male) from seven centers that received a specialist palliative care consultation during hospitalization were included.

**Results:**

In 37 (67.3%) of the 55 LVAD-centers, specialist palliative care was available. The median duration between LVAD-implantation and first specialist palliative care contact was 17 months (IQR 6.3–50.3 months). Median duration between consultation and death was seven days (IQR 3–28 days). 65% of consults took place in an intensive/intermediate care unit with half of the patients having a Do-Not-Resuscitate order. Care planning significantly increased during involvement (advance directives before: *n* = 15, after: *n* = 19, *p* < 0.001; DNR before: *n* = 20, after: *n* = 28, *p* < 0.001). Symptom burden as assessed at first specialist palliative care contact was higher compared to the consultation requests (request: median 3 symptoms (IQR 3–6); first contact: median 9 (IQR 6–10); *p* < 0.001) with a focus on weakness, anxiety, overburdening of next-of-kin and dyspnea. More than 70% of patients died during index hospitalization, one third of these in a palliative care unit.

**Conclusions:**

This largest European multicenter investigation of LVAD-patients receiving specialist palliative care shows a late integration and high physical and psychosocial symptom burden. This study highlights the urgent need for earlier integration to identify and address poorly controlled symptoms. Further studies and educational efforts are needed to close the gap between guideline-recommendations and the current status quo.

**Supplementary Information:**

The online version contains supplementary material available at 10.1186/s12904-024-01563-8.

## Background

The prevalence of heart failure is estimated around 2% in industrialized countries with increasing incidence caused by demographic changes [1]. Additionally, the survival of patients with heart failure improved over the last decades [1]. Thus, the number of patients with advanced heart failure who suffer from remaining symptoms despite optimal drug therapy is growing [2]. Besides heart transplantation, the implantation of left ventricular assist devices (LVAD) can be an effective therapeutic option [2, 3]. LVAD can be used during the waiting period for a donor heart (bridge to transplant concept, BTT). Due to the donor-organ shortage and the growing experience with these devices, LVAD are increasingly used as destination therapy (DT) and patients die with the device in place [4]. Special challenges and practical strategies for end-of life care in LVAD patients have been previously reported [5, 6].

The role of palliative care in outpatient heart failure care has long been neglected and a so called "death denying culture" was described for standard care [7]. Within the hospital setting, a difference between oncology and heart failure patients has also been shown [8]. In the latter, specialist palliative consults occurred significantly later and more often for advance care planning [8]. One reason for the lack of palliative care in this patient population is the difficult predictability of the disease course due to alternations between stable phases and sudden episodic decompensations [9]. Throughout the literature, positive effects of general (provided by all clinicians) as well as specialist (multi-professional team after training) palliative care in addition to standard care were described [3, 10, 11]. Palliative care is thus recommended as an integral part of the multidisciplinary team approach for patients with heart failure by American and European guidelines [2, 3, 11].

The integration of specialist palliative care in advanced heart failure patients with LVAD therapy was summarized before in a systematic review [12]. Almost all studies identified came from the United States and European data is scarce [12, 13]. A positive impact on the presence of care planning instruments, involvement of next-of-kin, reflection of treatment goals and end-of-life wishes, as well as symptom control and clinician satisfaction was observed [12].

This present study aims to explore specialist palliative care involvement in LVAD patients in a retrospective multicenter design in Germany. Specifically, we aim to (1) determine the extent to which LVAD centers offer and LVAD patients receive specialist palliative care, (2) describe the characteristics of patients who did receive specialist palliative care, (3) identify the timing, locations, and reasons for the involvement, and (4) outline the specific tasks performed by the palliative care teams.

## Methods

### Study design and ethics

A multicenter retrospective data collection was performed from January to December 2022. The study was approved by the local ethics committee in Duesseldorf before the start of the study (reference number: 2021–1600). For the additional centers, local requirements were followed and, if necessary, additional ethical approval was collected. Given the retrospective nature of our study, a waiver of informed consent was deemed appropriate based on the local ethics committee regulations. This study conforms to the principles outlined in the *Declaration of Helsinki*. Reporting of the study was performed according to the STROBE checklist [14].

### Study flow and data aggregation

According to the German heart surgery report, a total of 9,503 ventricular assist devices were implanted between 2012 and 2022 [15]. Based on information from the LVAD manufacturer Medtronic (Minneapolis, MN, USA) and Abbott (Illinois, IL, USA), there were 55 LVAD centers in Germany at the start of the study in October 2021 (Supplementary Table 1). Further investigation using the respective center’s website and short telephone calls was conducted to assess in which of these LVAD centers an additional specialist palliative care service was available (center-based data). Afterwards, these specialist palliative care centers were contacted via telephone and e-mail. The centers were asked about previous care for LVAD patients and the willingness to participate in the study. If both were applicable, the ethics regulations were organized (Supplementary Table 1). Thereafter, the centers received a link for digital data collection forms to enable paperless, data-protection-regulation (DSGVO) compliant and anonymized data transfer (Qualtrics, Provo, UT, USA). Forms were completed separately for each patient (patient-based data). Only patients ≥ 18 years with LVAD therapy that received a specialist palliative care consultation during hospitalization were included. Data collection included information on general patient characteristics, the consultation requests, and the setting and tasks of specialist palliative care involvement (Supplementary Fig. 1). For example, the palliative phase of illness at the initial specialist palliative care contact was evaluated using a tool that categorizes the condition into five levels: stable, unstable, deteriorating, dying, and deceased [16, 17]. Care planning instruments (advance directives, designated health care proxies and Do-Not-Resuscitate (DNR) orders) and symptoms (modified “Minimal Documentation System (MIDOS) for patients in palliative care” [18]) were specifically assessed.

### Data analysis

Data analysis was performed using Stata (Version 18, StataCorp LLC, College Station, TX, USA). Continuous data are presented as mean and standard deviation (SD) or median and interquartile range (IQR), as appropriate, and categorical data as frequencies (N) and proportions (%). To compare care planning instruments (existing, not-existing, missing information) before and after specialist palliative care involvement and also between the different LVAD concepts, χ2 tests for independence were used. For symptom comparisons, a complete case analysis was performed. After testing for normality with the Shapiro–Wilk method, the sum of symptoms (symptom burden) as assessed by the requesting physician and the specialist palliative care team were compared using a paired t-test. Given the small sample size, the McNemar's test was chosen to compare each specific symptom as assessed in the request and consult. Bonferroni correction was performed to adjust *p*-values (significance threshold *p* < 0.05) for multiple comparisons.

## Findings

### Study centers and patients

In 37 (67.3%) of 55 LVAD centers a specialist palliative care service was available. Thirteen of the specialist palliative care services indicated that they had not yet provided care for LVAD patients. 17 services did not answer our requests or declined to participate and could not be included (Supplementary Table 1). Accordingly, seven centers participated in this study.

### Patient characteristics

A total of 40 patients were included. Of these, 36 were men (90%) and the mean age was 65 years (youngest: 36 years; oldest 77 years). In 12 patients (30%), the treatment goal at implantation was heart transplantation (BTT), whereas 20 patients (50%) were treated with LVAD as destination therapy (DT). More than half of the patients (62.5%) required assistance with daily activities, 15% lived in a nursing facility. Further demographic data can be found in Table 1.

**Table 1:** Patient characteristics

Data	N (%) or mean (SD)
Sex, female	4 (10)
Age (years)	65.1 (7.9)
Underlying cardiac disease	
Ischemic cardiomyopathy	27 (67.5)
Dilatative cardiomyopathy	13 (32.5)
LVAD concept	
Bridge to transplant, BTT	12 (30)
Destination therapy, DT	20 (50)
Emergency implantation	3 (7.5)
Missing data	5 (12.5)
LVAD system	
HeartWare®	17 (42.5)
HeartMate II™	3 (7.5)
HeartMate 3™	20 (50)
Requirement of assistance with daily activities	
None	10 (25)
Existing	25 (62.5)
Missing data	5 (12.5)
ECOG activity index	
1 = able to carry out light work (housework/office)	3 (7.5)
2 = capable of all self-care; up > 50% of awake hours	2 (5)
3 = capable of limited self-care; confined to bed/chair > 50% of awake hours	13 (32.5)
4 = completely disabled and confined to bed/chair	19 (47.5)
missing data	2 (7.5)
Place of living before admission	
Alone	6 (15)
With next-of-kin	25 (62.5)
Nursing facility	6 (15)
Other	1 (2.5)
Missing data	2 (5)

*LVAD* Left Ventricular Assist Device, *ECOG* Eastern Cooperative Oncology Group activity index

### Specialist palliative care involvement

**Fig. 1:**
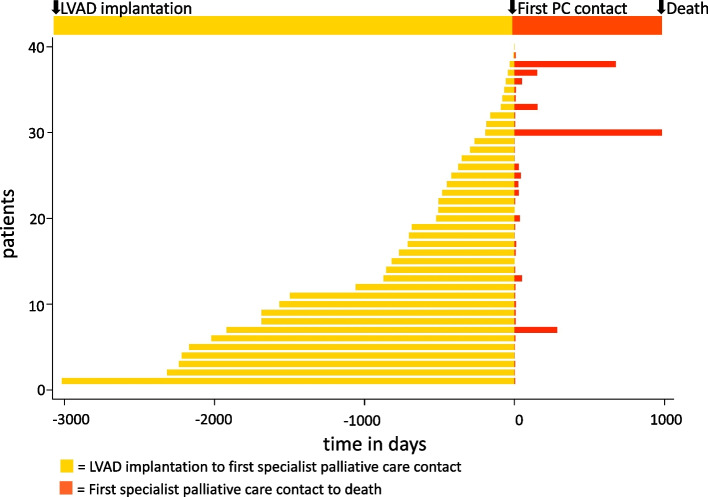
Time demonstrations. Duration from left ventricular assist device (LVAD) implantation to first specialist palliative care contact and duration from first contact to death in days. Patients are displayed in order of the duration of LVAD implantation to first specialist palliative care contact for visualization purposes

Overall, specialist palliative care requests were mainly made by cardiac surgeons (*n* = 20, 50%), followed by anesthesiologists (*n* = 6, 15%), cardiologists (*n* = 6, 15%), general practitioners (*n* = 3, 7.5%), general surgeons (*n* = 2, 5%) and others (*n* = 3). The median duration from LVAD implantation until first specialist palliative care contact (Fig. 1) was 17 months (IQR 6.3–50.3 months; minimum: 2 days; maximum: 8.3 years). 40% (*n* = 16) of first contacts occurred in the intensive care unit, whereas 27.5% (*n* = 11) took place on normal wards and 25% (*n* = 10) on intermediate care units (*n* = 3 missing data). According to the assessment of the palliative care physicians, patients were in the following palliative phases at initial contact [19]:

2 (5%) stable phase, 7 (17.5%) unstable phase, 20 (50%) deteriorating phase and 5 (12.5%) dying (missing data: 17.5%). During the index hospitalization, 19 patients (47.5%) died on an acute or intensive care ward, 10 (25%) on a palliative care unit. In total, 12 (30%) patients were transferred to a palliative care unit, two of them were discharged to a nursing home or another hospital, respectively. Further five (12.5%) patients were discharged home (one supported by a specialist palliative home care team) and two (5%) were transferred to another hospital. The median duration from first specialist palliative care contact to death (Fig. 1) was 7 days (IQR 3–28 days; minimum: 1 day; maximum: 2.7 years).

### Care planning instruments

After specialist palliative care integration, the number of existing advance directives (before: *n* = 15, 37.5%; after: *n* = 19, 47.5%; *p* < 0.001), designated health care proxies (before: *n* = 20, 50%; after: *n* = 23, 57.5%; *p* < 0.001), and DNR orders (before: *n* = 20, 50%; after: *n* = 28, 70%; *p* < 0.001) increased significantly (Table 2). At first specialist palliative care contact, existing care planning documents did not differ between LVAD concepts (DT vs BTT) (Table 2).

**Table 2:** Care instruments and specialist palliative care involvement. A: Before and after specialist palliative care involvement. B: Care planning documents before specialist palliative care involvement compared between the left ventricular assist devices (LVAD) concepts: bridge to transplant (BTT) or destination therapy (DT)

A	**Care instrument**	**Before**	**After**	**Test statistic**	***p-value***
	Advance directives	15 (37.50)	19 (47.50)	31.9336 ^a^	< 0.001*
	Health care proxy	20 (50)	23 (57.5)	18.0827 ^a^	< 0.001*
	DNR order	20 (50)	28 (70)	25.5238 ^a^	< 0.001*
**B**	**Care instrument**	**BTT**	**DT**	**Test statistic**	***p-value***
	Advance directives	3 (25)	9 (45)	4.1481 ^a^	0.126
	Health care proxy	6 (50)	11 (55)	1.7210 ^a^	0.423
	DNR order	2 (16.7)	12 (60)	5.7905^a^	0.055

Data are presented as N (%) for existing documents

*DNR* Do-Not-Resuscitate

^a^ Chi-square test for independence; *significant after Bonferroni correction (< 0.016)

### Symptoms

**Fig. 2:**
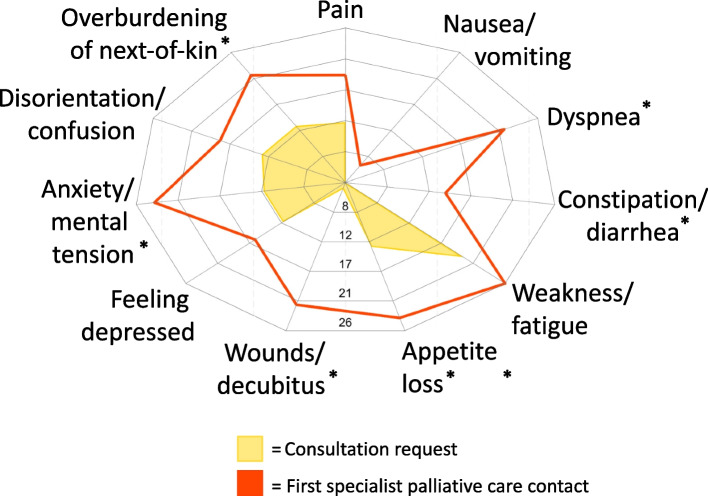
Symptom burden symptom intensities. Radar plot presenting the number of patients with each symptom documented in the consultation requests and in the notes from the first palliative care contact. * indicates a significant difference

**Fig. 3:**
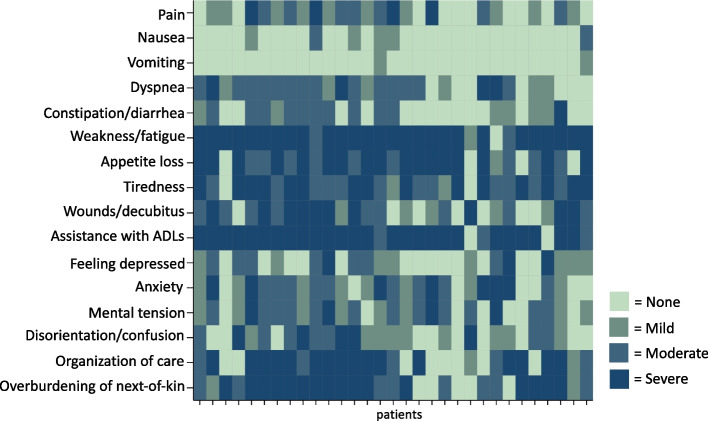
Symptom intensities. Heat map showing the intensity of the symptoms assessed at first specialist palliative care contact. Only patients with documented data are presented. ADLs, Activities of Daily Life

According to the information given in the consultation requests, weakness was the leading symptom which matches the specialist palliative care team assessment. However, the symptom burden identified by the specialist palliative care team was higher (Table 3). Figure 2 shows the number of patients with each symptom as documented by the requesting physician and by the specialist palliative care team after their first contact. Symptom intensities as assessed by the specialist palliative care team at first contact are visualized in Fig. 3. Information about symptom intensities of eight patients are missing due to sedation, weakness, dementia or language barriers. In six patients, complex respiratory symptoms were reported in the optional fill-in field. The main tasks for the specialist palliative care teams as mentioned in the notes were symptom control for respiratory symptoms specifically and during the deteriorating and dying phases as well as support for next-of-kin.

**Table 3:** Symptom assessment as documented in the consultation request and by the specialist palliative care team after their first contact in complete cases (*n* = 27)

Symptom	Request	First contact	Test statistic	*p*-value
Symptom burden	3 (3–6)	9 (6–10)	-8.2106 ^a^	< 0.001*
Pain	12 (44.4)	19 (70.4)	5.44 ^b^	0.02
Nausea/vomiting	3 (11.1)	6 (22.2)	1.29 ^b^	0.257
Dyspnea	10 (37)	22 (81.5)	12.00 ^b^	< 0.001*
Constipation/diarrhea	3 (11.1)	14 (51.9)	11.00 ^b^	< 0.001*
Weakness	20 (74.1)	27 (100)	7.00 ^b^	0.008
Appetite loss	13 (48.2)	24 (88.9)	11.00 ^b^	< 0.001*
Wounds/decubitus	4 (14.8)	22 (81.5))	16.2 ^b^	< 0.001*
Feeling depressed	12 (44.4)	16 (59.3)	1.6 ^b^	0.206
Anxiety/tension	12 (44.4)	24 (88.9)	12.00 ^b^	< 0.001*
Disorientation/confusion	13 (48.2)	18 (66.7)	5.00 ^b^	0.025
Overburdening of next-of-kin	13 (48.2)	22 (81.5)	9.00 ^b^	0.003*

Data is presented as median (interquartile range) or N (%)

^a^ Paired t-test; ^b^ McNemar’s test; *significant after Bonferroni correction (< 0.005)

## Discussion

### Main findings

This multicenter retrospective study provides insights into both center- as well as patient-based characteristics in specialist palliative care for patients treated across German LVAD centers. While 67% of LVAD centers in Germany offer specialist palliative care, at least 13 of these 37 specialist palliative care services have not previously provided care for LVAD patients. Half of the 40 included patients were treated with LVAD as a DT. The time from LVAD implantation to first specialist palliative care contact varied from two days to more than eight years. In contrast, the median duration from first specialist palliative care contact to death was seven days, but also varied between one day and almost three years. In 65%, the first specialist palliative care contact occurred in intensive or intermediate care units and 63% of patients were in a deteriorating or dying phase. 73% of all patients died during the index hospitalization with first specialist palliative care contact. Care planning instruments were present in less than half of the critically ill patients and over the time of specialist palliative care involvement, the number of planning instruments increased significantly. At palliative care consultation request, 50% of patients had a DNR order. The symptom focus was weakness, anxiety and tension, overburdening of next-of-kin and dyspnea. The specialist palliative care teams assessed higher symptom burden compared to the requesting physicians, especially regarding dyspnea, constipation and appetite loss. Symptom intensities were severe for weakness and overburdening of next-of-kin as well as in the organization of care and assistance with activities of daily life.

### What this study adds

Over the last decade, around 650 to 1,000 LVAD have been implanted annually in Germany [15]. Within our study, we included 40 LVAD patients who had received specialist palliative care, although we invited all available specialist palliative care services within German LVAD centers. Overall, compared to the number of LVAD implanted per year, this number of patients seems relatively low. In a case series from Germany and Switzerland, 11% of all patients received palliative care with center specific variance [13]. In the United States, between 2006 and 2014, 4% of LVAD patients received palliative care with 7.2% in 2014 [20]. The DNR status appeared to be a strong predictor (adjusted odds ratio: 28.30) for palliative care consultations [20]. In our study, 50% of patients had a DNR status before specialist palliative care involvement was requested. This underscores the prevalent perception of palliative care necessity when no other treatment options are available, reflecting the “death-denying culture” among cardiologists described in the introduction.

The first specialist palliative care contact occurred long after LVAD implantation, but shortly before death (Fig. 1). None of the included patients had a pre-LVAD specialist palliative care consultation or involvement during the LVAD consideration. The late integration of specialist palliative care in the LVAD process observed in our study is in line with previous data that resulted in American and European guideline recommendations for early integration of palliative care in the LVAD course [21–24]. According to these, preparedness planning for decision making and advance care planning should be performed prior to the LVAD implantation [22, 23]. A recently published qualitative study highlighted the impactful experience of an LVAD implantation regarding the patients’ values as well as personal goals and priorities [25]. In our study, around 50% of patients had advance directives and designated health care proxies before the first palliative care consultation, which was significantly increased after specialist palliative care involvement. Advance care planning should be focused not only by palliative care but also by primary treating physicians, e.g. cardiac surgeons, cardiologists or intensivists. Understanding the impact, like notably reduced deaths within the intensive care unit following advance care planning by the specialist palliative care team, has the potential to influence current practices [26, 27].

In our study, 12 patients were transferred to the palliative care unit, of whom ten died there. A qualitative interview study investigated the perspectives of bereaved caregivers on the end-of-life experience of patients with LVAD. A high level of confusion through the perception of lacking knowledge and comfort in LVAD care by the palliative care teams was reported [28]. Case reports, experiences and clinical protocols can give guidance to clinicians [13, 23, 29–31].

Our study reveals a discrepancy in symptom assessment between the requesting physicians and the first assessment by the specialist palliative care team. This might also explain the underuse of specialist palliative care in this patient cohort, as requesting clinicians might underestimate symptom burden. A study that assessed palliative care need by a screening tool for heart failure patients in the regular LVAD outpatient clinic showed that 67% of patients were in need for palliative care [32]. Our results on symptom prevalence and intensity are mostly consistent with those of Strangl and colleagues [32]. The primary focus of symptoms is weakness (80% in Strangl and colleagues; 78% in our study) with elevated rates of moderate or high intensities. While in Strangl and colleagues study pain was reported as being the leading physical symptom, only 26% of patients in our cohort experienced pain. Both, intensive and specialist palliative care clinicians, should be trained in knowledge and skills to provide sufficient symptom control in this special group of patients.

Overall, our results indicate that specialist palliative care in LVAD patients is underutilized and occurs rather late, which is in concordance with previous data. Although early integration before LVAD implantation is recommended, barriers like limited specialist palliative care resources, infrastructure (one third of German LVAD centers lack a palliative care service) and the patients, next-of kin and clinicians attitude towards palliative care need to be studied.

An optimal and feasible time point for integration needs to be assessed. Along this way, explorations like this present study raise the attention on this urgent topic.

### Strengths and limitations of the study

Given the retrospective nature of our study, several limitations apply. The process of data collection is described in detail, however we might not have reached all LVAD centers and specialist palliative care services. Due to the high number of centers that declined to participate, there may be a risk of selection bias. No conclusive assessment of the specialist palliative care prevalence in LVAD patients in Germany was performed. Information on the total number of LVAD cases performed at the included centers is not available. Furthermore, no comparison between LVAD patients with and without specialist palliative care can be drawn based on our data. There was a relatively high amount of missing data that cannot be addressed because of the retrospective data collection from medical records. Lastly, the composition and resources of the specialist palliative care teams differ across included centers and might influence their involvement in the treatment of LVAD patients. Overall, the involvement of specialist palliative care observed in this study conducted in Germany may not be extended to other countries.

## Conclusion

This first multicenter study on specialist palliative care involvement in LVAD patients in Europe shows an underutilized and late integration. Two thirds of LVAD centers offer specialist palliative care, however the first contact with the specialist palliative care team often occurs shortly before death and in the intensive care unit. The specialist palliative care team assessed a significantly higher symptom burden compared to the requesting physicians with a focus on weakness, anxiety, overburdening of next-of-kin and dyspnea. Our study sheds light on the gap between the status quo of specialist palliative care in German LVAD centers and the European and American recommendations. Further studies and educational efforts are essential to improve integration timing as well as symptom control in this patient population.

## Supplementary Information

Supplementary Material 1: Supplemental Table 1: LVAD centers and available specialist palliative care centers. Supplemental Fig. 1: Survey for data collection in English (translated) and German (original version).

## Data Availability

The data that support the findings of this study are not openly available due to reasons of sensitivity and are available from the corresponding author upon reasonable request.

## Abbreviations

LVAD: Left Ventricular Assist Device
BTT: Bridge To Transplant
DT: Destination Therapy
DNR: Do Not Resuscitate

## Availability of data and materials

The datasets used and analyzed during the current study are available from the corresponding author on reasonable request.
