# Supplementary material for: Multicenter exploration of specialist palliative care in patients with left ventricular assist devices – a retrospective study
Source: BMC Palliat Care. 2024 Sep 23;23:229. doi: 10.1186/s12904-024-01563-8 (PMC11421205; doi:10.1186/s12904-024-01563-8)
Supplement: Supplementary file 1 — Supplementary Material 1: Supplemental Table 1: LVAD centers and available specialist palliative care centers. Supplemental Fig. 1: Survey for data collection in English (translated) and German (original version). [file 12904_2024_1563_MOESM1_ESM.docx]

**SUPPLEMENTARY MATERIAL:**

**Multicenter exploration of specialist palliative care in patients with
left ventricular assist devices – a retrospective study**

**Theresa Tenge^1,2^, Shaylin Shahinzad^2^, Stefan Meier^1^, Manuela Schallenburger^2^, Yann-Nicolas Batzler^2^, Jacqueline Schwartz^2^, Anja Coym^3^, Johannes Rosenbruch^4^, Mitra Tewes^5^, Steffen T Simon^6^, Carmen Roch^7^, Ute Hiby^8^, Christian Jung^9^, Udo Boeken^10^, Jan Gaertner^11,12^, Martin Neukirchen^1,2^**

1. Department of Anesthesiology, Medical Faculty and University Hospital Duesseldorf, Heinrich Heine University Duesseldorf, Germany
2. Interdisciplinary Center for Palliative Medicine, Medical Faculty and University Hospital Duesseldorf, Heinrich Heine University Duesseldorf, Center for Integrated Oncology Aachen Bonn Cologne Duesseldorf (CIO ABCD), Germany
3. Palliative Care Unit, Department of Oncology, Hematology and Bone Marrow Transplant, University Medical Center Hamburg-Eppendorf, Hamburg, Germany
4. Department of Palliative Medicine, LMU University Hospital, LMU Munich, Munich, Germany
5. Department of Palliative Medicine, University Hospital Essen, University of Duisburg-Essen, Essen, Germany
6. Department of Palliative Medicine and Center for Integrated Oncology Aachen Bonn Cologne Duesseldorf
   (CIO ABCD), University of Cologne, Faculty of Medicine and University Hospital, Cologne, Germany
7. Interdisciplinary Center for Palliative Medicine, University Hospital Wuerzburg, Wuerzburg, Germany
8. Palliativstation, Campus Bad Neustadt, RHÖN-Klinikum AG, Bad Neustadt an der Saale, Germany
9. Department of Cardiology, Pulmonology and Vascular Medicine, Medical Faculty and University Hospital Duesseldorf, Heinrich-Heine-University Duesseldorf, Germany
10. Department of Cardiac Surgery, Medical Faculty and University Hospital Duesseldorf, Heinrich-Heine-University Duesseldorf, Germany
11. Palliative Care Center Basel, Basel, Switzerland
12. Department of Clinical Research, University of Basel, Basel, Switzerland

Table of content

1. **Supplementary Table 1:** Left ventricular assist device (LVAD) centers in Germany with respective available specialist palliative care services and information on study inclusion (ethics committee information) or reason for non-inclusion............................................ 2
2. **Supplementary Figure 1:** Survey for data collection in English (translated) and German (orginal version)................................................................................................................. 5

**Supplementary Table 1:** Left ventricular assist device (LVAD) centers in Germany with respective available specialist palliative care services and information on study inclusion (ethics committee information) or reason for non-inclusion.

| **LVAD center** | **City** | **Specialist palliative care service** 0=no;  1 = yes | **Included** 1 = yes | **Ethics statement** | **Reason non-inclusion**  1 = no answer/no interest;  2 = no patients |
| --- | --- | --- | --- | --- | --- |
| Universitätsklinikum Aachen | Aachen | 1 |  |  | 1 |
| Klinikum Augsburg | Augsburg | 1 |  |  | 1 |
| Herz- und Gefäßzentrum Bad Bevensen | Bad Bevensen | 0 |  |  | 2 |
| Kerckhoff-Klinik Bad Nauheim | Bad Nauheim | 0 |  |  | 2 |
| Rhön-Klinikum AG | Bad Neustadt | 1 | 1 | Approved by Bavarian ethics committee  Reference: 2022-1096  Date: May 25^th^ 2022 |  |
| Herz- und Diabeteszentrum NRW | Bad Oeynhausen | 0 |  |  | 2 |
| Schüchtermann-Klinik | Bad Rothenfelde | 0 |  |  | 2 |
| Segeberger Kliniken Bad Segeberg | Bad Segeberg | 0 |  |  | 2 |
| Deutsches Herzzentrum Berlin | Berlin | 1 |  |  | 2 |
| BG Universitätsklinikum Bergmannsheil | Bochum | 0 |  |  | 2 |
| Klinik und Poliklinik für Herzchirurgie | Bonn | 1 |  |  | 2 |
| Klinikum Braunschweig | Braunschweig | 1 |  |  | 2 |
| Sana-Herzzentrum Cottbus | Cottbus | 0 |  |  | 2 |
| Herzzentrum Dresden | Dresden | 0 |  |  | 2 |
| Herzzentrum Duisburg | Duisburg | 1 |  |  | 2 |
| Universitätsklinikum Düsseldorf | Düsseldorf | 1 | 1 | Approved by ethics committee  Reference: 2021-1600  Date: August 6^th^ 2021  Amendment approved: March 13^th^ 2023 |  |
| Universitätsklinikum Erlangen | Erlangen | 1 |  |  | 1 |
| Universitätsklinikum Essen | Essen | 1 | 1 | Approved by ethics committee  Reference: 23-11436-BO  Date: September 12^th^ 2023 |  |
| Universitäts Klinikum Frankfurt | Frankfurt | 1 |  |  | 1 |
| Universitäts-Herzzentrum Freiburg Bad Krozingen | Freiburg | 1 |  |  | 1 |
| Uniklinikum Giessen/ Marburg | Giessen | 1 |  |  | 1 |
| Universitätsmedizin Göttingen | Göttingen | 1 |  |  | 2 |
| Universitätsklinikum Halle (Saale) | Halle | 0 |  |  | 2 |
| Asklepios Klinik St. Georg | Hamburg | 1 |  |  | 1 |
| Asklepios Klinikum Harburg | Hamburg | 1 |  |  | 2 |
| Universitatsklinikum Hamburg-Eppendorf | Hamburg | 1 | 1 | Approval by ethical committee  Reference: 2022-200494-BO-bet  Date: Sept 9^th^ 2022 |  |
| Medizinische Hochschule Hannover | Hannover | 1 |  |  | 2 |
| UniversitätskKlinikum Heidelberg | Heidelberg | 1 |  |  | 1 |
| Universitätsklinikum des Saarlandes | Homburg | 1 |  |  | 2 |
| Universitätsklinikum Jena | Jena | 1 |  |  | 1 |
| Klinikum Karlsburg | Karlsburg | 0 |  |  | 2 |
| Helios Klinik für Herzchirurgie | Karlsruhe | 0 |  |  | 2 |
| Klinikum Kassel | Kassel | 1 |  |  | 1 |
| Universitätsklinikum Schleswig-Holstein | Kiel | 1 |  |  | 1 |
| Herzzentrum der Uniklinik Köln | Köln | 1 | 1 | Positive statement after reviewing ethical approval from University Duesseldorf |  |
| Helios Klinikum Krefeld | Krefeld | 1 |  |  | 1 |
| MediClin Herzzentrum Lahr/ Baden | Lahr/ Baden | 0 |  |  | 2 |
| Herzzentrum Leipzig | Leipzig | 0 |  |  | 2 |
| Universitaetsklinikum Schleswig-Holstein | Lübeck | 1 |  |  | 1 |
| Universitätsklinikum Magdeburg A.ö.R. | Magdeburg | 0 |  |  | 2 |
| Deutsches Herzzentrum München | München | 0 |  |  | 2 |
| Klinikum der LMU München Großhadern | München | 1 | 1 | Approved by Bavarian ethics committee  Reference: 2022-1096  Date: May 25^th^ 2022 |  |
| Universitätsklinikum Münster | Münster | 1 |  |  | 1 |
| Klinikum Nürnberg (Süd) | Nürnberg | 1 |  |  | 2 |
| Klinikum Oldenburg | Oldenburg | 1 |  |  | 1 |
| Universitätsklinikum Regensburg | Regensburg | 1 |  |  | 2 |
| University Rostock | Rostock | 1 |  |  | 1 |
| Herz-Kreislaufzentrum Rotenburg an der Fulda | Rotenburg an der Fulda | 0 |  |  | 2 |
| Helios Klinikum Siegburg | Siegburg | 0 |  |  | 2 |
| Robert-Bosch-Krankenhaus GmbH | Stuttgart | 1 |  |  | 2 |
| Universitätsklinikum Tübingen | Tübingen | 1 |  |  | 2 |
| Universitätsklinikum Ulm | Ulm | 1 |  |  | 2 |
| SHG Kliniken Völklingen | Völklingen | 1 |  |  | 2 |
| Helios Universitätsklinikum Wuppertal | Wuppertal | 1 |  |  | 1 |
| Universitätsklinikum Würzburg | Würzburg | 1 | 1 | Approved by ethics committee  Reference:2022-0704 01  Date: Nov. 23^th^ 2022 |  |

**Supplementary Figure 1:** Survey for data collection in English (translated) and German (orginal version).

English version:

Interdisciplinary Center for Palliative Medicine (IZP) - University Hospital Düsseldorf - Moorenstraße 5 - 40225 Düsseldorf

**DATA SHEET**

Clinic:

Patient No.:

**Patient characteristics**

**Age:** _______________

**Gender:** Male ☐ Female ☐ Diverse ☐

**Underlying cardiac disease:**

☐ Ischemic Cardiomyopathy

☐ Dilated Cardiomyopathy

☐ Other:____________________

**LVAD concept**

☐ Bridge to Transplant (BTT): HTX Done? ☐ Change of therapy goal to DT? ☐

☐ Destination Therapy (DT)

☐ Emergency LVAD

**LVAD model**

☐ HeartWare

☐ HeartMate II

☐ HeartMate III

☐ Other: _________________

**ECOG/ Karnofsky Index: Score for the assessment of the general condition**

☐ ECOG (0-5): ______

☐ Karnofsky (100-0): ______

**Barthel Index** (0-100): Index for the assessment of a person's disability or everyday abilities.

☐ At first contact ___________

☐ At discharge: __________

**Degree of care requirement**

☐ Existing

☐ None

**Housing**

☐ Alone (home) ​

☐ With relatives (home)

☐ Nursing facility

☐ Other: _____________

**Need**

**Question according to the consultation request:**

_____________________________________________________________________________________

**Previous course of therapy according to the consultation request:**

_____________________________________________________________________________________

**Symptoms according to the consultation request:**

_____________________________________________________________________________________

**Requesting physician**

☐ Cardiac surgeon

☐ Anesthesiology

☐ General Surgery

☐ Nursing, if so, which discipline:

☐ Others, if so, which ones: _________

**Palliative stage**

☐ Stable phase

☐ Instable phase

☐ Deteriorating phase

☐ Dying

**Discharge**

☐ To palliative care unit; ☐ waiting list?

☐ Deceased

☐ Home

☐ Another hospital

☐ Others: _____________

**General conditions**

**Temporal aspects**

Duration of inpatient stay (date of admission – date of discharge/death/transfer): ______________

Date of consultation request: __________________________

Date of first specialist palliative care consultation: ___________

Date LVAD Implantation: ____________________________

Date of death (follow-up may be necessary): ___________

**Location of first specialist palliative care contact**

☐ Normal ward

☐ Intensive Care Unit

☐ Intermediate Care Station

☐ Others: _____________

**Next-of-kin present?**

☐ Yes

☐ No

☐ Unclear

**Needs**

**Consultation request – current problems:**

a. Pain ☐ yes ☐ no

b. Nausea/vomiting ☐ yes ☐ no

c. Shortness of breath ☐ yes ☐ no

d. Constipation/ diarrhea ☐ and ☐ no

e. Weakness ☐ yes ☐ no

f. Lack of appetite ☐ yes ☐ no

g. Care problems wounds/ decubitus ☐ yes ☐ no

h. Depression ☐ yes ☐ no

i. Anxiety/ tension ☐ yes ☐ no

j. Disorientation/confusion ☐ yes ☐ no

k. Excessive next-of kin burden ☐ yes ☐ no

l. Other problems: _______________________________________

**Symptoms at first specialist palliative care contact**

a. Pain ☐ none ☐ light ☐ medium ☐ strong

b. Nausea ☐ none ☐ light ☐ medium ☐ strong

c. Vomiting ☐ none ☐ light ☐ medium ☐ strong

d. Shortness of breath ☐ none ☐ light ☐ medium ☐ stark

e. Constipation ☐ none ☐ light ☐ medium ☐ stark

f. Weakness ☐ none ☐ light ☐ medium ☐ strong

g. Lack of appetite ☐ none ☐ light ☐ medium ☐ strong

h. Fatigue ☐ none ☐ light ☐ medium ☐ strong

i. Care problems wounds/ decubitus ☐ none ☐ light ☐ medium ☐ stark

j. Need for help with activities of daily life ☐ none ☐ light ☐ medium ☐ stark

k. Depression ☐ none ☐ light ☐ medium ☐ strong

l. Anxiety ☐ none ☐ light ☐ medium ☐ strong

m. Mental tension ☐ none ☐ light ☐ medium ☐ strong

n. Disorientation/ Confusion ☐ none ☐ light ☐ medium ☐ strong

o. Problems in the organization of care ☐ none ☐ light ☐ medium ☐ strong

p. Excessive next-of kin burden ☐ none ☐ light ☐ medium ☐ strong

q. other problems/symptoms ______________________________________

**Care planning**

Prior to the consultation request

Advance directive: ☐ yes ☐ no

Health care proxy: ☐ yes ☐ no

Do-not-resuscitate order (DNR) ☐ yes ☐ no

After palliative care contact

Advance directive: ☐ yes ☐ no

Health care proxy: ☐ yes ☐ no

Do-not-resuscitate order (DNR) ☐ yes ☐ no

Original German version:

Interdisziplinäres Zentrum für Palliativmedizin (IZP) - Universitätsklinikum Düsseldorf - Moorenstraße 5 - 40225 Düsseldorf

**ANALYSEBOGEN**

Klinik:

Patient:in Nr.:

1. **Patientenmerkmale**

**Alter:** _______________

**Geschlecht:** Männlich ☐ Weiblich ☐ Divers ☐

**Kardiologische Grunderkrankung:**

☐ Ischämische Kardiomyopathie

☐ Dilatative Kardiomyopathie

☐ Weitere: ____________________

**LVAD Konzept**

☐ Bridge to Transplant (BTT): HTX erfolgt? ☐ Therapiezielwechsel zu DT? ☐

☐ Destination Therapy (DT)

☐ Notfall LVAD

**LVAD model**

☐ HeartWare

☐ HeartMate II

☐ HeartMate III

☐ weiteres: _________________

**ECOG/ Karnofsky Index: Score zur Beurteilung des Allgemeinzustandes**

☐ ECOG (0-5): ______

☐ Karnofsky (100-0): ______

**Barthel Index** (0-100): Index for the assessment of a person's disability or everyday abilities.

☐ Bei Erstkontakt: __________

☐ Bei Entlassung: __________

**Pflegegrad**

☐ Keiner

☐ Ja

**Wohnort**

☐ Allein (home)

☐ mit Angehörigen (home)

☐ Heim

☐ Sonstiges: _____________

1. **Bedarf**

**Fragestellung laut Konsilanforderung:**

_____________________________________________________________________________________

**Bisheriger Therapieverlauf laut Konsilanforderung:**

_____________________________________________________________________________________

**Symptomlast:**

_____________________________________________________________________________________

**Fachrichtung des Antragstellers**

☐ Herzchirurgie ​

☐ Anästhesiologie

☐ Allgemeinchirurgie

☐ Pflege, wenn ja, welche Fachdisziplin:

☐ Sonstige, wenn ja, welche: _________

**Palliativstadium**

☐ Rehabilitationsphase

☐ Frühe Terminalphase

☐ SpäteTerminalphase

☐ Finalphase

**Verlegung**

☐ auf die Palliativstation; ☐ Warteliste?

☐ verstorben

☐ nach Hause

☐ weiteres Krankenhaus

☐ Sonstiges: _____________

1. **Rahmenbedingungen**

**Zeitliche Aspekte**

Dauer des stationären Aufenthaltes (Datum Aufnahme – Datum Entlassung/ Versterben/ Verlegung): ______________

Datum der Konsilanforderung: __________________________

Datum des palliativmedizinischen Erstkontaktes: ___________

Datum LVAD Implantation: ____________________________

Sterbedatum (evtl. Nachverfolgung notwendig): ___________

**Ort des Erstkontaktes**

☐ Normalstation

☐ Intensivstation

☐ Intermediate Care Station

☐ Sonstiges: _____________

**Angehörige anwesend?**

☐ Ja

☐ Nein

☐ Unklar

1. **Bedürfnisse**

**Konsilanforderung – aktuelle Probleme:**

a. Schmerzen ☐ ja ☐ nein

b. Übelkeit/ Erbrechen ☐ ja ☐ nein

c. Luftnot ☐ ja ☐ nein

d. Verstopfung/ Durchfall ☐ ja ☐ nein

e. Schwäche/ Müdigkeit ☐ ja ☐ nein

f. Appetitlosigkeit ☐ ja ☐ nein

g. Pflegeprobleme Wunden/ Dekubitus ☐ ja ☐ nein

h. Depressivität ☐ ja ☐ nein

i. Angst/Anspannung ☐ ja ☐ nein

j. Desorientiertheit/ Verwirrtheit ☐ ja ☐ nein

k. Überforderung des sozialen Umfelds ☐ ja ☐ nein

l. Andere Probleme: _______________________________________

**MIDOS bei Aufnahme**

a. Schmerz ☐ keine ☐ leicht ☐ mittel ☐ stark

b. Übelkeit ☐ keine ☐ leicht ☐ mittel ☐ stark

c. Erbrechen ☐ keine ☐ leicht ☐ mittel ☐ stark

d. Luftnot ☐ keine ☐ leicht ☐ mittel ☐ stark

e. Verstopfung ☐ keine ☐ leicht ☐ mittel ☐ stark

f. Schwäche ☐ keine ☐ leicht ☐ mittel ☐ stark

g. Appetitmangel ☐ keine ☐ leicht ☐ mittel ☐ stark

h. Müdigkeit ☐ keine ☐ leicht ☐ mittel ☐ stark

i. Pflegeprobleme Wunden/ Dekubitus ☐ keine ☐ leicht ☐ mittel ☐ stark

j. Hilfebedarf bei Aktivitäten des tgl. Lebens ☐ keine ☐ leicht ☐ mittel ☐ stark

k. Depressivität ☐ keine ☐ leicht ☐ mittel ☐ stark

l. Angst ☐ keine ☐ leicht ☐ mittel ☐ stark

m. Anspannung ☐ keine ☐ leicht ☐ mittel ☐ stark

n. Desorientiertheit/ Verwirrtheit ☐ keine ☐ leicht ☐ mittel ☐ stark

o. Probleme bei der Organi- sation der Versorgung ☐ keine ☐ leicht ☐ mittel ☐ stark

p. Überforderung der Familie ☐ keine ☐ leicht ☐ mittel ☐ stark

q. andere Probleme/ Symptome ______________________________________

**Vorsorgeplanung**

bei Konsilanforderung

Patientenverfügung: ☐ ja ☐ nein Vorsorgevollmacht: ☐ ja ☐ nein

Betreuung: ☐ ja ☐ nein DNR ☐ ja ☐ nein

nach palliativmedizinischem Kontakt

Patientenverfügung: ☐ ja ☐ nein Vorsorgevollmacht: ☐ ja ☐ nein

Betreuung: ☐ ja ☐ nein DNR: ☐ ja ☐ nein
